# Diarrheagenic *Escherichia coli* Phylogroups Are Associated with Antibiotic Resistance and Duration of Diarrheal Episode

**DOI:** 10.1155/2015/610403

**Published:** 2015-02-25

**Authors:** Susan Mosquito, Maria J. Pons, Maribel Riveros, Joaquim Ruiz, Theresa J. Ochoa

**Affiliations:** ^1^Instituto de Medicina Tropical Alexander von Humboldt, Universidad Peruana Cayetano Heredia, Lima 031, Peru; ^2^ISGlobal, Barcelona Centre for International Health Research (CRESIB), Hospital Clínic, Universitat de Barcelona, 08036 Barcelona, Spain; ^3^Universidad Peruana de Ciencias Aplicadas (UPC), Lima 09, Peru; ^4^School of Public Health, University of Texas, Houston, TX 77030, USA

## Abstract

Conventionally, in *Escherichia coli*, phylogenetic groups A and B1 are associated with commensal strains while B2 and D are associated with extraintestinal strains. The aim of this study was to evaluate diarrheagenic (DEC) and commensal *E. coli* phylogeny and its association with antibiotic resistance and clinical characteristics of the diarrheal episode. Phylogenetic groups and antibiotic resistance of 369 *E. coli* strains (commensal strains and DEC from children with or without diarrhea) isolated from Peruvian children <1 year of age were determined by a Clermont triplex PCR and Kirby-Bauer method, respectively. The distribution of the 369 *E. coli* strains among the 4 phylogenetic groups was A (40%), D (31%), B1 (21%), and B2 (8%). DEC-control strains were more associated with group A while DEC-diarrhea strains were more associated with group D (*P* < 0.05). There was a tendency (*P* = 0.06) for higher proportion of persistent diarrhea (≥14 days) among severe groups (B2 and D) in comparison with nonsevere groups (A and B1). Strains belonging to group D presented significantly higher percentages of multidrug resistance than the rest of the groups (*P* > 0.01). In summary, DEC-diarrhea strains were more associated with group D than strains from healthy controls.

## 1. Introduction

Conventionally,* Escherichia coli*, a common isolate in clinical laboratories, is classified into two major groups: commensal and pathogenic. Additionally pathogenic isolates may produce different diseases, being then subdivided in diarrheogenic and extraintestinal* E. coli*. Human infections caused by extraintestinal* E. coli* include meningitis, urinary tract infections, sepsis, pneumonia, surgical site infections, and infections in other extraintestinal locations [1]. However, when classified into subtypes,* E. coli* mainly fall into four phylogenetic groups: A, B1, B2, and D [1]. Previous studies have shown that commensal* E. coli* strains tend to be associated within phylogenetic groups A and B1 [1, 2], whereas the extraintestinal pathotypes fall within phylogenetic groups B2 and D [3, 4]. Regarding uropathogenic* E. coli* (UPEC) strains determinants including phylogroups markers are well established. However, for diarrheagenic* E. coli* (DEC), the scenario remains unclear. There is no information about the association between the phylogenetic group and the clinical data of the diarrheal episode [5].

Previous reports describe the emerging antibiotic resistance in commensal and diarrheagenic* E. coli* in Peru [6–10]. However, there is no sufficient data in the correlation of phylogeny and antibiotic resistance [11]. Therefore we conducted this study to determine the association between the phylogenetic group and antibiotic resistance in a large number of* E. coli* (commensal and diarrheagenic) strains from Peruvian infants.

## 2. Materials and Methods

### 2.1. Samples

Commensal and diarrheagenic* E. coli* strains were isolated during a previous passive surveillance diarrhea study [12]. In this study 1032 children were followed from 2 to 12 months of life, obtaining a total of 1079* E. coli* strains that were analyzed by a real time multiplex PCR to determine DEC pathotypes [13]. A total of 369 isolates from this study were randomly selected and analyzed, including 74 commensal* E. coli*, 94 DEC from asymptomatic children (DEC-control), and 201 DEC isolated from children with diarrhea (DEC-diarrhea). DEC pathotypes included in this study were enteropathogenic* E. coli* (EPEC), enterotoxigenic* E. coli* (ETEC), enteroaggregative* E. coli* (EAEC), and diffusely adherent* E. coli* pathotypes (DAEC); Shiga toxin-producing* E. coli* (STEC) and enteroinvasive* E. coli* (EIEC) strains were not included due to their low prevalence [12].

### 2.2. Phylogenetic Group Determination

The phylogenetic groups were determined as previously described [14]. In all cases the bacteria DNA was extracted boiling.

### 2.3. Clinical Data of Diarrheal Episodes

Variables such as episode duration (days), maximum number of stools per day, total number of stools per episode, and a modified Vesikari modified score [15] were analyzed and associated with phylogenetic groups in those cases in which no coinfections were previously reported [12].

### 2.4. Antibiotic Resistance

Antibiotic susceptibility to ampicillin (10 *μ*g), trimethoprim-sulfamethoxazole (23.75/1.25 *μ*g), chloramphenicol (30 *μ*g), nalidixic acid (30 *μ*g), and tetracycline (30 *μ*g) was determined by disk diffusion in accordance with the CLSI guidelines [16]. Multiresistance was defined as resistance to three or more unrelated antibiotic families.

### 2.5. Statistical Analysis

Vesikari severity score was expressed by mean ± standard deviation and median (range) values were given for duration of the episode and number of stools. The comparisons between groups were made using chi-squared or Fisher's exact test. Student's *t*-test was used for the comparison of Vesikari severity scores between groups.

## 3. Results 

### 3.1. Phylogenetic Group Frequency

The* E. coli* strains (DEC and commensals) were distributed in the four phylogenetic groups: A (147 isolates, 40%), D (116 isolates, 31%), B1 (76 isolates, 21%), and B2 (30 isolates, 8%). No significant difference in the prevalence of phylogenetic groups was found within each pathotype when analyzed by control/diarrhea. In total were analyzed 87 EPEC (38 DEC-control, 49 DEC-diarrhea), 83 ETEC (26 DEC-control, 57 DEC-diarrhea), 94 EAEC (24 DEC-control, 70 DEC-diarrhea), 31 DAEC (6 DEC-control; 25 DEC-diarrhea), and 74 commensal isolates.

**Figure 1:**
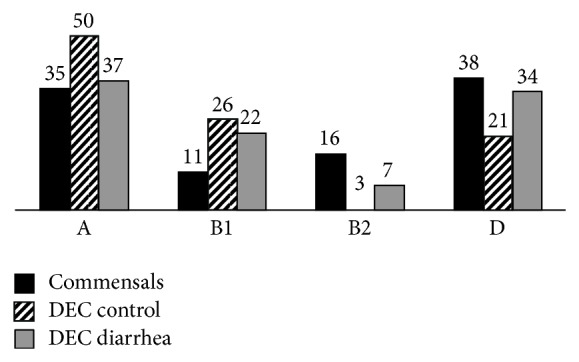
Percentage of phylogenetic groups in commensal* E. coli* strains (*n* = 74), diarrheagenic* E. coli* from healthy controls (DEC-control) (*n* = 94), and DEC from children with diarrhea (DEC-diarrhea) (*n* = 201).

The phylogroup A was the most common, in both groups, control and diarrhea, in EPEC (45 isolates, 52%) and ETEC (44, 53%), while mostly DAEC isolates (27 isolates, 87%) belong to phylogroup D. Regarding EAEC, differences were found between those isolates causing diarrhea and those recovered from healthy children. Thus EAEC isolates causing diarrhea were mostly classified as phylogroup D (29 isolates, 41%), while those recovered from healthy children predominantly belong to the phylogroup A (10 isolates, 42%) (Table 1). Analyzing together the DEC isolates, those classified as DEC-control strains were more associated with A group (50%) while the DEC-diarrhea strains were more associated with D group (34%) (*P* < 0.05). Meanwhile, commensal* E. coli* (*n* = 74) were more associated with A (26 isolates, 35%) and D (28 isolates, 38%) phylogroups. The commensal group also had a high prevalence of B2 group (12 isolates, 16%) unlike both DEC groups (*P* < 0.05). Both DEC-control (24 isolates, 26%) and DEC-diarrhea (54 isolates, 27%) groups were more associated with the phylogroup B1 than commensals strains (7 isolates, 14%) (*P* < 0.05) (Figure 1).

### 3.2. Clinical Data of Diarrheal Episodes

From the 201 DEC-diarrhea isolates analyzed in the study, 127 strains were isolated from diarrhea episodes in which no other pathogen was detected. In these 127 patients, no significant differences were found for the studied variables among the four phylogenetic groups. In general, the episode duration was 5 days (1–25), the maximum number of stools/day was 5 (3–11), the total number of stools/episode was 20 (3–128), and the Vesikari score was 6 ± 2.6. We found a higher proportion (*P* = 0.06) of persistent diarrhea (14 or more days) among B2-D groups (23.9%) compared to among A-B1 groups (9.88%). No differences were found for either acute or prolonged diarrhea (7–14 days) between severe (B2-D) and nonsevere (A-B1) groups.

### 3.3. Antibiotic Resistance

**Figure 2:**
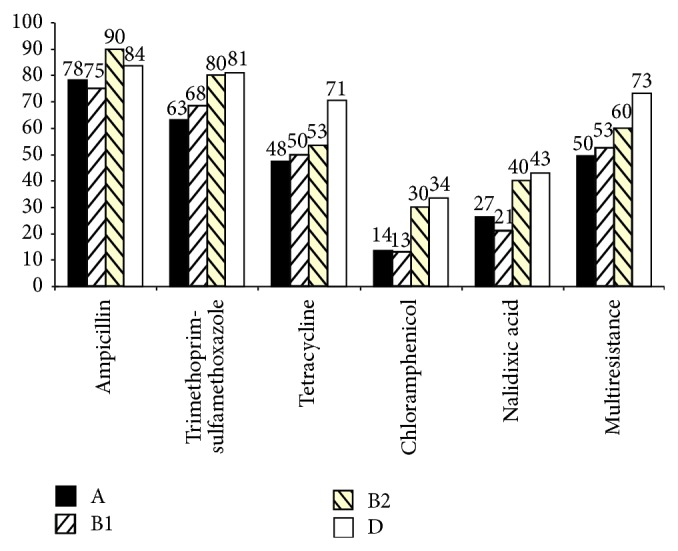
Percentages of multiresistance (resistance to 3 or more different antibiotics families) and antibiotic resistance among the four phylogenetic groups A (*n* = 147), B1 (*n* = 76), B2 (*n* = 30), and D (*n* = 116).

Resistance to trimethoprim-sulfamethoxazol, tetracycline, chloramphenicol, and nalidixic acid and multiresistance were significantly different among the four phylogenetic groups (*P* < 0.05) (Figure 2). In general, B2 and D groups presented higher percentage of antibiotic resistance than A and B1 groups. In the case of multiresistance D group presented significantly higher percentages than the rest of the groups (*P* < 0.01) (Figure 2).

## 4. Discussion

The* E. coli* phylogroups differ in their ecological niches, life-history characteristics, and propensity to cause disease. In this manner B2 and D groups are less frequently isolated from the environment [17]. Regarding human illness,* E. coli* isolates recovered from extraintestinal body sites are more likely to belong to B2 or D phylotypes than to A or B1 [18, 19]. However, differences in the prevalence of the different phylogenetic groups among virulent extraintestinal* E. coli* have been observed in previous studies [20]. Despite the fact that the two phylogenetic groups most frequently related with virulence in the extraintestinal* E. coli* are the aforementioned B2 and D, some reports have shown a high frequency of group A (46%) among* E. coli* causing urinary infections [20]. Alternatively, some reports showed that gut commensal* E. coli* were mostly related with group A [14]. In addition, in a previous study in children in Costa Rica, commensal* E. coli* were related to phylogenetic groups A and D (36%), while the studied DEC belong to B1 (35%), A (29%), B2 (23%), and D (14%) [19]. Another study analyzing DEC (EAEC, EPEC, and STEC) isolated from children in Rumania showed that 51% of the strains belonged to group A, followed by 23% of the strains that belonged to group B2 [21]. In the present study, commensal strains were more associated with A and D groups as has been previously reported [19].

Phylogenetic groups B2 and D have been related with virulence factors that cause infections at an extraintestinal level. Additionally, the B2 strains have been shown to persist for longer periods in infants than other* E. coli* strains [22].

Previous studies have tried to relate the clinical data of infection with the phylogenetic groups [23]. However, these types of studies have not addressed intestinal pathogenic strains that cause diarrhea. In this report, no significant differences were found for the clinical variables analyzed among the four phylogenetic groups. However, we found a greater tendency of persistent diarrhea in phylogenetic groups B2 and D (24%) than in groups A and B1 (10%). Regarding isolates belonging to the B2 group, this long persistence has been previously observed [22], while no data has been found regarding other phylogroups. In this line an association between the diarrheagenic pathotype DAEC and persistent diarrhea was also observed, in accordance with what has been previously reported [24]. Interestingly, DAEC isolates belong largely to the D phylogroup.

When we analyzed the relation between phylogenetic group and antibiotic resistance we found that the isolates belonging to the group D were more related with multiresistance than those belonging to other groups. Although previous reports in extraintestinal strains showed that virulence-related phylogenetic groups, especially B2, were associated with low levels of antibiotic resistance [11] our data showed a different scenario, in which B2 and D isolates were those exhibiting high levels of antibiotic resistance. A possibility to take into account is the possibility that as phylogroup D results in more prolonged diarrhea, the use of antibacterial agents may be needed, and then these isolates may be under a more intense antibiotic pressure which may facilitate the acquisition of antibiotic resistance mechanisms.

DEC accounts for more than 120,000 deaths/year, being involved, together with rotavirus in around 40% of all diarrhea related children deaths [25]. Recent studies have showed that both EPEC and ETEC isolates are related with an increased mortality [26]; in fact, it is considered that ETEC isolates account for more than 40,000 deaths each year. In this sense it is of interest to note that in the present study most of the ETEC or EPEC isolates belong to the phylogroup A, classically considered as a low-virulent phylogroup. Studies on extraintestinal* E*.* coli* have showed that the development of antibiotic resistance may be correlated with a decreased virulence [27]. Thus, although no clear reason may be stated to explain the high percentage of DEC isolates belonging to low-virulent phylogroups, the high levels of antibiotic resistance present in the area and these described inverse relations between virulence and antibiotic resistance may be suggested as a potential explanation.

One study limitation is the fact that specific virulence factors encoding genes were not studied; therefore, a direct relationship between virulence and resistance was not possible to evaluate. Furthermore, multilocus sequence typing (MLST) method is more specific than the Clermont triplex PCR method used in this study [28]. However, the Clermont triplex PCR method is a rapid and cost-effective method that has been used extensively. Moreover, this method is able to be implemented in resource constraining sites. A recent study indicates that strains belonging to cryptic lineages of* Escherichia* are the more related to failure by this triplex method; however, in human fecal samples these lineages are unlikely to be found (2-3% frequency) [29].

## 5. Conclusion

The present data show the relationships between* E. coli* phylogenetic lineages, diarrheagenic character, and severity of illness. Moreover, an association between phylogroup D and prolonged disease duration has been found. Interestingly, the isolates belonging to this phylogroup also presented the higher levels of multiresistance, showing that those isolates able to cause a more prolonged disease also possess higher levels of antibiotic resistance, probably because they usually required antibiotic treatment and then are under a more intense antibiotic pressure.

Further investigations to elucidate the relationship between phylogeny, specific virulence factors, and mechanisms of resistance are needed in order to better understand DEC and commensal* E. coli* strains.

## Figures and Tables

**Table 1:** 

Phylogroup	Commensal		Diarrheagenic pathotypes															
			EAEC (94)				EPEC (87)				DAEC (31)				ETEC (83)			
			Control		Diarrhea		Control		Diarrhea		Control		Diarrhea		Control		Diarrhea	
	*N*	%	*N*	%	*N*	%	*N*	%	*N*	%	*N*	%	*N*	%	*N*	%	*N*	%
A	26	35.1	10	41.7^*^	20	28.6^*^	21	55.3	24	49.0	0	0	2	8.0	16	61.5	28	49.1
B1	8	10.8	4	16.7	14	20.0	12	31.6	11	22.4	0	0	0	0	8	30.8	19	33.3
B2	12	16.2	2	8.3	7	10.0	0	0	5	10.2	1	16.7	1	4.0	0	0	2	3.5
D	28	37.8	8	33.3	29	41.4	5	13.1	9	18.4	5	83.3	22	88.0	2	7.7	8	14.0

Total	74	100	24	100	70	100	38	100	49	100	6	100	25	100	26	100	57	100

^*^ *P* < 0.05.
